# Optimizing the Pharmacotherapy of Vascular Surgery Patients at Hospital Admission and Discharge (PHAROS): Protocol for a Quasi-Experimental Clinical Uncontrolled Trial

**DOI:** 10.2196/60728

**Published:** 2025-03-19

**Authors:** Slavka Porubcova, Kristina Lajtmanova, Kristina Szmicsekova, Veronika Slezakova, Jan Tomka, Tomas Tesar

**Affiliations:** 1 Department of Organisation and Management of Pharmacy Faculty of Pharmacy Comenius University Bratislava Slovakia; 2 Hospital Pharmacy The National Institute of Cardiovascular Diseases Bratislava Slovakia; 3 Department of Pharmacology Faculty of Pharmacy Comenius University Bratislava Slovakia; 4 Department of Vascular Surgery The National Institute of Cardiovascular Diseases Bratislava Slovakia

**Keywords:** pharmacotherapy, hospital pharmacy, vascular surgery, patient safety, risk reduction, pharmacist-proposed interventions

## Abstract

**Background:**

Patient safety is essential in pharmacotherapy, especially in surgical contexts, due to the elevated risk of drug-related complications. Vascular surgery patients are particularly susceptible because of their complex medication needs and underlying health conditions. Improved safety monitoring and targeted pharmaceutical care in collaboration with physicians are crucial to minimize these risks and enhance patient outcomes.

**Objective:**

This protocol evaluates whether structured pharmaceutical care interventions—including medication reconciliation, medication review, and patient education—can reduce the prevalence of drug-related problems at hospital admission and discharge in vascular surgery patients.

**Methods:**

This prospective, uncontrolled study was conducted over 1 year in the Vascular Surgery Department at the National Institute of Cardiovascular Diseases in Bratislava, Slovakia. The study included adult patients with carotid artery disease or lower extremity artery disease who were on 3 or more medications, with an estimated sample size of approximately 100 patients. The primary intervention involved 3 key changes in practice: medication reconciliation at both admission and discharge, where hospital pharmacists review and verify medication lists; medication review to identify and address drug-related problems; and patient education at discharge. Pharmacist-proposed interventions were documented and communicated to the physician for treatment adjustments. The primary outcome is the change in drug-related problem prevalence from hospital admission to discharge. Secondary outcomes include the acceptance rate of pharmacist recommendations and patient understanding of pharmacotherapy. Data collection involved documenting the number, type, and frequency of drug-related problems; the anatomical therapeutic chemical classification of medications associated with drug-related problems; and patients’ social, demographic, and clinical characteristics, with a focus on factors related to drug-related problems, comorbidities, and medication use. Data analysis will use the paired Wilcoxon signed-rank test to compare the prevalence of drug-related problems and medication counts between admission and discharge. Continuous variables will be presented as means (SDs), while categorical variables will be reported as counts and percentages. Patient understanding of pharmacotherapy will be evaluated using a 3-point scale, classifying understanding as good (2-3 points per medication), modest (1-2 points), or poor (0-1 point).

**Results:**

Recruitment began in September 2021 and concluded in August 2022. Data collection occurred continuously during hospital stays, capturing demographics, comorbidities, pharmacotherapy, and drug-related problems at admission and discharge. Important milestones included the initial data review, which began in August 2023 to assess recruitment and data quality, including an early evaluation of drug-related problems. The primary analysis was completed in January 2024, focusing on the reduction in drug-related problems, intervention acceptance, and patient understanding. The final report was to be prepared by June 2024, disseminating the findings on pharmacist-led intervention impacts.

**Conclusions:**

This study should demonstrate that pharmacist-led interventions in collaboration with physicians can reduce pharmacotherapy risks and optimize medicine management for patient safety.

**Trial Registration:**

ClinicalTrials.gov NCT04930302; https://clinicaltrials.gov/study/NCT04930302

**International Registered Report Identifier (IRRID):**

RR1-10.2196/60728

## Introduction

### Background

Pharmaceutical care was first defined in 1990 by Hepler and Strand [1] as “the responsible provision of drug therapy for the purpose of achieving definite outcomes that improve a patient’s quality of life.” Worldwide, pharmaceutical care is currently considered a patient-centered approach, replacing the previous product orientation (dispensing medications) [2,3]. The pharmacist actively cooperates not only with the patient but also with health care professionals in health promotion, disease prevention, evaluation, monitoring, and adjustment and initiation of drug use in order to ensure an effective and safe drug regimen, achieve positive clinical results, and reduce the economic costs of care [2,4].

Currently, patient safety in health care delivery is at the forefront of interest worldwide [5,6]. Patient safety is the absence of preventable harm to a patient during the process of providing health care and reduction of the risk of unnecessary harm associated with health care to an acceptable minimum. Hospital pharmacists [4,7] can make a significant contribution to the safe, effective, and rational use of medicines by hospitalized patients, especially high-risk medication and look-alike and sound-alike medications, through their close surveillance as well as advising on the most appropriate use of medicines [7]. Identification of drug-related problems (DRPs) and proposal of solutions for DRPs by hospital pharmacists is a tool to ensure safe and effective pharmacotherapy for patients [8,9].

The Pharmaceutical Care Network Europe Association (PCNE) defines a DRP as a problem, event, or circumstance related to pharmacotherapy that affects or has the potential to affect a desired therapeutic effect [10]. An example is the arbitrary withdrawal of metformin by patients due to persistent diarrhea [11]. The PCNE has developed a classification system as a tool to accurately identify DRPs. The current version is V9.00 as of 2019 [10]. This classification tool is used throughout our study to ensure consistency in identifying and categorizing DRPs.

DRPs are generally all problems related to the use of a drug [10]. DRPs include adverse drug reactions (ADRs), an unintended reaction after medication administration [11-14], medication errors, and any phenomenon that may lead to improper use of a drug under the control of a health care professional or patient [15-17].

Although it is not common practice in Slovakia to report all DRPs, this is not an issue unique to the country. Many nations face similar challenges, especially where comprehensive pharmacovigilance systems are still developing [18]. In 2019, only 1128 suspected ADRs were reported, of which only 8% were reported from pharmacists. Overall, up to 26% of reports were classified as severe (ie, required hospitalization of the patient or caused permanent harm to the patient). Although state authorities have seen an increase in the number of spontaneous reports, their number probably does not correspond to their actual occurrence. Currently, we do not have statistically evaluated health care costs due to medication errors in Slovakia [18].

We distinguish between intentional and unintentional DRPs [10,19,20]. There is no uniform classification of the severity of DRPs. It is necessary to focus on the identification and elimination of unintentional DRPs.

At the same time, according to current statistics, the average age of the population is increasing worldwide, which is directly related to the higher prevalence of polymorbidity and polypharmacy in the population [21-23].

There are certain indicators on the basis of which an increased incidence of unintentional DRPs can be expected. In a hospital environment, these are the high number of drugs, which is related to low adherence, potential interactions, accumulation of medication errors, more frequent hospitalizations, and increased treatment costs [24-27]; a low level of understanding of the therapy being used among patients [28-30]; older age of patients [21-23,28]; and absence of caregivers for older adult patients [29].

These indicators may be associated with the incidence of treatment errors related to primary care.

### Prior Work

Pharmacists use several strategies to reduce DRPs, including medication reconciliation (MedRec), medication review (MedRev), and patient education [30-34]. Prescription errors during hospital admission are common, with studies showing that these errors occur in up to 67% of patients [35]. MedRec has been shown to identify at least one DRP in 60% of patients, with 18% being clinically significant [36]. The incidence of drug discrepancies during hospital transitions, especially at discharge, is high, with some studies showing significant discrepancies upon patient discharge [37,38]. According to a 2020 Organization for Economic Co-operation and Development report, pharmacy-led MedRec before discharge reduces drug discrepancies and the associated risks to patient health [39]. A meta-analysis conducted by Mekonnen et al [40] in 2016 found that pharmacist-led MedRec reduces hospital readmissions by 67%, emergency admissions by 28%, and hospitalizations by 19%.

MedRec involves obtaining a best possible medication history (BPMH) from various sources, including medical records, community pharmacies, and patient interviews. This is compared with the patient’s current medication list to identify discrepancies, which are then discussed with health care professionals and resolved [32-34,41,42]. MedRev further optimizes pharmacotherapy by eliminating unnecessary medications, adjusting dosages, or addressing drug interactions [43].

Studies on pharmaceutical interventions typically focus on specific patient groups, such as those with cardiovascular diseases, renal failure, or older adult patients. For example, Stemer et al [44] identified 487 DRPs in 138 visits, with a 54.7% acceptance rate for pharmacist recommendations. Hohn et al [45] found a rate of 0.41 unintentional medication errors per patient in vascular surgery patients. In patients with chronic renal insufficiency, pharmacist interventions improved renal function, particularly in more severe cases [46]. Older adult patients also showed positive outcomes, with pharmacist interventions reducing hospital readmissions by 16% and hospitalization rates due to DRPs by 80% [47].

Overall, pharmacist-led interventions are effective in reducing DRPs, improving pharmacotherapy, and enhancing patient outcomes. However, the types and numbers of DRPs detected vary across studies, indicating the need for standardized definitions in research on DRPs [19,33,48].

### Trial Objectives

This study aims to assess the impact of pharmaceutical care in collaboration with physicians on the prevalence of DRPs at hospital admission and discharge in patients with carotid artery disease or lower extremity artery disease hospitalized in the Department of Vascular Surgery. These patients often experience polypharmacy, making them particularly suitable for pharmacotherapy optimization through pharmacist-led interventions. MedRec, MedRev, and patient education provided by pharmacists are new interventions at our hospital and were not part of the standard of care prior to the study.

The key focus area of this project is the identification of DRPs, their occurrence, and their type. As part of further research, we want to analyze the degree of acceptance of the proposed changes in pharmacotherapy by physicians, document the Anatomical Therapeutic Chemical Classification (ATC) groups of drugs with the highest incidence of DRPs, and identify patients at highest risk for DRPs taking into consideration their personal and health information.

### Trial Hypotheses

The null hypothesis is that pharmaceutical care provided at hospital admission and hospital discharge does not reduce the prevalence of DRPs in patients with carotid artery disease or lower extremity artery disease hospitalized in the Department of Vascular Surgery.

The alternative hypothesis is that pharmaceutical care provided at hospital admission and hospital discharge reduces the prevalence of DRPs in patients with carotid artery disease or lower extremity artery disease hospitalized in the Department of Vascular Surgery.

## Methods

### Study Design

This study is a single-center, prospective, uncontrolled biomedical clinical trial conducted over 1 year. The study took place in a hospital setting, specifically in the Department of Vascular Surgery at the National Institute of Cardiovascular Diseases in Bratislava, Slovakia. The intervention included MedRec, MedRev, and patient education, all performed by a trained pharmacist following the High 5s Project Standard Operating Protocol for Medication Reconciliation [24] and PCNE guidelines [10]. All pharmacist recommendations and proposed changes to therapy were documented in writing, recorded in the patient’s medical record, and communicated to the attending physician.

Our study followed the Standards for Quality Improvement Reporting Excellence (SQUIRE) guidelines [49].

### Study Population

Adult (≥18 years of age) vascular surgery patients with carotid artery disease or lower extremity artery disease admitted for hospitalization at the study setting during the course of the study were recruited.

#### Inclusion Criteria

To participate, patients needed to be ≥18 years old at the date of admission for hospitalization, taking at least 3 medications administrated systematically, and have carotid artery disease or lower extremity artery disease.

#### Exclusion Criteria

Patients were excluded for any of the following reasons: admitted for an acute condition; transferred from other hospitals or wards; not willing to sign the informed consent form for the study; not understanding the Slovak language; had any mental disorder affecting memory and recall ability (such as Alzheimer disease); any other reason, at the investigator’s discretion, why he or she deemed the participant not eligible for study participation (all such reasons were recorded); or participating in another clinical study.

### Study Sample Size

To calculate the sample size, we aimed to accept a type I error rate with a *P* value ≤.05. The study seeks to achieve 80% power to detect a small effect size of 0.3 using the Wilcoxon signed-rank test. Based on these assumptions, the required sample size was calculated as 94 patients. To account for potential dropouts, the sample size was increased to 120 patients. The sample size calculation was performed using G*Power software version 3.1 [50]. Similar studies published in this field have comparable sample sizes [51].

### Data Sources and Measurements

Data were drawn from the hospital information system (HIS), medical and nursing reports, patient interviews, and contacting outpatients.

### Primary Outcomes

The primary outcome is a change in the prevalence of DRPs at hospital admission versus hospital discharge.

### Secondary Outcomes

The secondary outcomes are the acceptance rate of the pharmaceutical intervention by physicians and patient understanding of their pharmacotherapy.

### Variables

The variables collected include the number, type, and frequency of DRPs; ATC of drugs causing DRPs; medical, social, and demographic characteristics of patients; health condition of patients; comorbidities; and patient understanding of their pharmacotherapy assessed on a 3-point scale at hospital admission.

We assessed the patients’ basic social, demographic, and clinical characteristics, with a particular emphasis on evaluating their pharmacotherapy and the incidence of DRPs. Additionally, we examined the degree of acceptance of pharmacists’ recommendations by physicians and the outcomes of DRP resolutions. Our analysis will also identify the medications most frequently associated with the occurrence of DRPs. Patients’ understanding of their pharmacotherapy was also be evaluated.

### Time Points for Intervention

#### At Hospital Admission

##### Current Condition at Patient Admission

A vascular surgery patient with carotid artery disease or lower extremity artery disease comes for a planned hospitalization with a report from the attending physician, with or without an internal preoperative examination. The physician, in cooperation with the nurse, examines the patient on admission, draws information from the patient’s medical records, and in the case of rehospitalization, from the HIS and internal preoperative examination. The physician will prepare an admission report, which will record the patient’s previous illnesses, the current state of health, the reason for hospitalization, and all associated illnesses. Patients often bring a list of medications they are taking; the physician will consult with the patient on the completeness of this list. He or she detects then records possible allergies to drugs and food and other forms of intolerance or allergic manifestations. He or she is interested in the use of addictive substances, alcohol, and drugs of abuse and the frequency of their use. The patient signs informed consent that he or she consents to hospitalization and treatment. The admission report, which also includes the drug course (current drug record, which is updated once a day; if necessary, it is possible to insert notes, consultation examinations, orders for laboratory tests, and other information), will be prepared both in the HIS and in printed form. Each hospitalized patient has a printed medical record that is more comprehensive and contains more detailed information than the HIS records. It contains all the patient’s health results, required examinations, daily drug courses, all daily updated nursing records, records of the patient’s diet, and more. Based on the prescription of medications in the course, the nurse prepares and administers medications to the patient, and a sudden change in pharmacotherapy by the physician is reported to the nurse orally then recorded in the course. Some patients keep some of their medication with themselves and dose it themselves according to the physician’s instructions.

##### MedRec by Pharmacists at Patient Admission

The patient is normally admitted to a planned hospitalization by the physician in cooperation with the nurse, as aforementioned. If the patient is older than 18 years, takes more than 3 medicines, speaks and understands the Slovak language, and has signed informed consent to participate in biomedical research, after being placed in a bed, a pharmacist comes to the patient’s bedside and performs a MedRec. During the MedRec, the pharmacist creates a record of the patient’s therapy, the original of which is placed in the patient’s medical record, and a copy is placed in the pharmacist’s records.

The steps in the MedRec involved the following. As part of the invitation for the hospitalization (by telephone or in writing), the patient was asked to bring all their medications and a complete medication list, which was used in consultation with the pharmacist regarding their proper use. The BPMH was completed.

The source of information can be a drug record, an admission report, hospital records, historical records from the HIS, or information from the patients or their family member. A BPMH is different and more complex than the routine history of primary treatment (which is often a rapid history of patient treatment). The BPMH includes the name of the medicine, dose, and frequency and administration route of the medicines that the patient is currently taking, although it may differ from what was actually written in the drug list.

The types of drugs that need to be recorded in the BPMH include prescription drugs, over-the-counter drugs, nutritional supplements, herbal medicines, medicinal teas, recreational drugs, and regular consumption of certain foods (eg, grapefruit). Special emphasis should be placed on specific forms of medicines, such as inhalers, eye drops, topical semisolid medicines, or medicines taken every few weeks (bisphosphonates).

One of the recorded parameters is patient understanding. This is evaluated in 3 steps and scored as 0 or 1: The patient knows (1) or does not know (0) the name of the drug, the patient knows (1) or does not know (0) the indication of the drug, and the patient knows (1) or does not know (0) the dosage of the drug. The 3-point scale was based on previously published studies by Cline et al [52], Boonstra et al [53], and Marfo et al [54], who evaluated patients’ knowledge based on their understanding of the drug name, dosage, duration of treatment, indication, relationship of the drug to food, duration of therapy, or route of administration. If the patient was unable to attend the interview, other sources were used to obtain a medical history or to clarify conflicting information. Other resources should never be a substitute for a thorough conversation with the patient or family members.

Regarding verification and documentation of the BPMH, the BPMH list should be verified by more than one other source. Sources for the initial acquisition of an overview of pharmacotherapy are the admission report, the history of hospitalization, outpatient reports, HIS, and the course at admission.

According to the World Health Organization standard operating procedure, a retroactive MedRec model was used for our biomedical research. In a retroactive model, in accordance with the aforementioned method, the patient is admitted by a physician in cooperation with a nurse by default, and a drug course—a daily prescription of drugs for the patient – is created. In this case, the BPMH is determined after admission by a physician or nurse.

The result of the MedRec is a comparison of medicines prescribed to and actually used by patients (BMPH) with marked discrepancies.

During the process of obtaining the BPMH, the pharmacist informs patients to always share their doubts about the correct use of the medication with the medical staff.

##### MedRev With Pharmaceutical Intervention at Patient Admission

The basis for patient therapy optimization is the acquisition of the BPMH and patient factors, such as the reason for hospitalization, current health status, comorbidities, height, weight, heart rate, blood pressure, and the results of examinations of biochemical and hematological parameters.

The detected BPMH is written in the case report form. It is then analyzed in the context of the patient’s overall health condition, and DRPs are identified. Each detected discrepancy is assigned an alphanumeric code according to the PCNE V9.00 classification. The patient’s personal data are anonymized.

Discrepancies in therapy should be consulted with the treating physician within 24 hours of admission.

##### Evaluation of Therapy Based on DRPs and PCNE Classification

After receiving the BPMH and a detailed study of the reason for hospitalization, current medical condition, and any patient comorbidities, the pharmacist collects information about the patient’s weight, height, heart rate, and blood pressure from the admission report. Subsequently, the results of biochemical and hematological examinations are studied. The pharmacist focuses on the results of examinations related to pharmacotherapy and the determination of the function of elimination organs. These are mainly the levels of serum potassium, serum creatinine, serum uric acid, liver transaminases, lipidogram, and C-reactive protein. Renal function is calculated based on the Cockcroft and Gault creatinine clearance estimate.

##### Duplicate Treatment

The pharmacist controls duplication in the drugs used, considering the use of the same substance in 2 drugs, use of the same substance in 2 dosage forms, use of 2 substances from the same pharmacological group, and double inhibition of the renin-angiotensin-aldosterone axis by angiotensin-converting enzyme inhibitors and angiotensin II receptor blockers.

Duplicity is recorded in written form in the final evaluation of the patient’s pharmacotherapy; duplication is discussed with the patient, who confirms or refutes the actual use of the duplicate drugs, and the most appropriate procedure is proposed in collaboration with the attending physician.

##### Indications of Used Drugs and Contraindications

The pharmacist checks the indications of the drugs used according to the patient’s comorbidities. The pharmacist checks whether all comorbidities are treated according to evidence-based medicine and according to treatment procedures developed by local, national, or international authorities. The result of this step is the identification of missing drugs in the patient’s pharmacotherapy.

Furthermore, drugs whose use has no clear indication are identified. In older adult patients (≥65 years), the pharmacist also considers the use of potentially inappropriate drugs. The pharmacist follows the EU (7)-PIM list [55], which was designed for patients aged 65 years and older. The result of this step is the identification of drugs that do not have a clear indication or are inappropriate due to the patient’s age.

The pharmacist also checks whether the prescribed drug is appropriate for the specific patient.

Drugs that the patient should and should not take, drugs whose indications are not clearly known from the available data, and contraindicated drugs are recorded in the final evaluation of the patient’s pharmacotherapy, in cooperation with the attending physician; subsequently, the most appropriate procedure is proposed.

##### Drug Management

The pharmacists verify compliance with maximum recommended doses, considering patient-specific factors, and records any excess in the pharmacotherapy evaluation. They assess the suitability of dosage forms, administration practices (including infusion components, timing, and food interactions), and screen for potential drug interactions using LexiComp and other resources, documenting any clinically significant findings. All observations and recommendations are reviewed with the attending physician to determine the appropriate course of action.

##### Adverse Drug Reactions

If a newly manifested ADR not documented in the patient’s medical history is suspected, the pharmacist evaluates the degree of causality between the drugs used and the manifestations of the ADR, which is proven on the basis of laboratory parameters or the patient’s subjective complaint. The pharmacist reports suspicions of ADR to the State Institute of Drug Control via an electronic form available from [56].

ADRs are recorded in the final evaluation of the patient’s pharmacotherapy.

The individual DRP findings shall be entered in the form as an appropriate code according to the PCNE classification [10].

The result of the pharmacist’s intervention at patient admission is the completion of the MedRec form at admission and creation of an accurate list of medicines taken by the patient. All comments on pharmacotherapy with the proposed solutions are written in the form of a summary report and undergo consultation with the physician.

#### At Hospital Discharge

##### Current Condition at Patient Discharge

When discharging a patient, the attending physician evaluates the patient’s state of health and prepares a discharge report with complete information about the procedures that the patient underwent during hospitalization. The discharge report also includes the current results of the patient’s laboratory examinations, an overview of current pharmacotherapy, and recommendations to the patient’s general practitioner. The nurse explains the regimen measures in relation to his or her state of health to the patient and gives the patient medication for the 3 days following discharge.

##### MedRec by Pharmacists at Patient Discharge

The pharmacist performs MedRec when discharging a patient from the hospital, similar to the process at admission. When evaluating a patient’s pharmacotherapy on discharge from hospital, the BPMH obtained at the patient’s admission will be used as a source of information. The BPMH is then compared with the list of medicines that are recorded in the release report. The pharmacist will compare 2 lists of medicines, focusing on the identification of DRPs with special regard to the re-introduction of the chronic therapy the patient was taking before hospitalization. As part of this research, the pharmacist met with the patient during his or her discharge from the hospital and discussed with him or her the management of further pharmacotherapy. Pharmacists provided an understandable summary list of a patient’s medicines explaining the importance and the correct use of the medicine.

##### MedRev With Pharmaceutical Intervention at Patient Discharge

The pharmacist analyzes the pharmacotherapy in the context of the patient’s state of health and the results of laboratory tests, similar to the patient’s admission, as described in the previous sections. Detected DRPs are then consulted with the treating physician and recorded in the case report form.

The result of the pharmacist’s intervention at discharge is the completion of the MedRec form for discharge and the documentation of all comments on pharmacotherapy in the form of a summary report. Discrepancies and comments are consulted with the physician.

**Figure 1:**
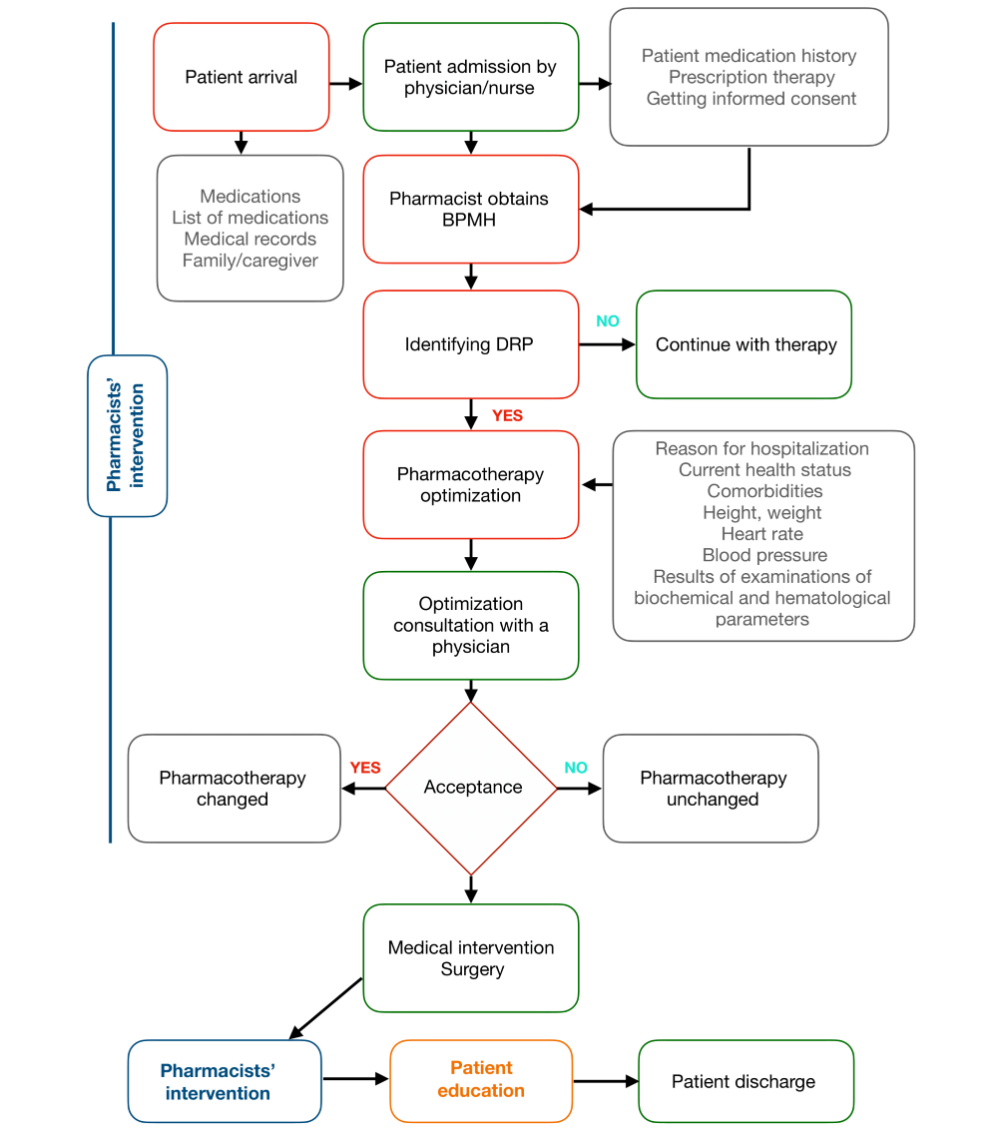
Procedure scheme.

The complete scheme of the procedure can be seen in Figure 1.

### Ethical Considerations

#### Human Subject Ethics Review Approvals

The study was approved by the Ethics Committee of the National Institute of Cardiovascular Diseases (approval number 1625/21; May 26, 2021) and registered on ClinicalTrials.gov (trial registration number NCT04930302; June 16, 2021). All research procedures adhered to the ethical standards of the institutional and national research committees and were conducted in accordance with the 1964 Helsinki Declaration and its later amendments or comparable guidelines.

#### Informed Consent

Written informed consent was obtained from all participants before any research-related activities were initiated. The consent form, approved by the Ethics Committee, was provided in Slovak to ensure participants’ understanding. By signing the consent form, participants consented to the publication and presentation of their pseudonymized data.

#### Privacy and Confidentiality

Patients’ personal data were anonymized to ensure confidentiality. The Participant Identification Sheet was securely stored at the study center, alongside other documentation. Anonymized data were entered into the case report form in the MIA DMS online database, which operates within Europe.

#### Compensation Details

No compensation was provided to participants for their involvement in this study.

## Results

This protocol outlines the planned study design and anticipated timeline for the PHAROS trial, aimed at evaluating the impact of pharmacist-led interventions on DRPs in patients undergoing vascular surgery. The key stages of the trial and their anticipated completion dates are described in the following sections.

### Study Initiation and Recruitment

Patient recruitment began in September 2021 and was completed in **A**ugust 2022. The target sample size of 100 patients was reached.

### Data Collection

Data collection occurred continuously during each patient’s hospital stay, including assessments at admission and discharge to document patient demographics, comorbidities, pharmacotherapy details, and DRPs. These data will help evaluate the prevalence and types of DRPs, intervention acceptance rates, and changes in patient understanding of pharmacotherapy.

### Analysis and Reporting Milestones

For the initial data review, preliminary data analyses began in August 2023 to assess recruitment efficacy and data integrity. This phase includes an initial assessment of DRPs and intervention types recorded during patient admission.

The primary analysis, including full data analysis encompassing outcomes related to DRP reduction, intervention acceptance, and patient pharmacotherapy understanding, was completed in January 2024.

For the final report and dissemination, the study findings, including the impact of pharmacist interventions on patient outcomes, were compiled and prepared for dissemination by June 2024.

**Figure 2:**
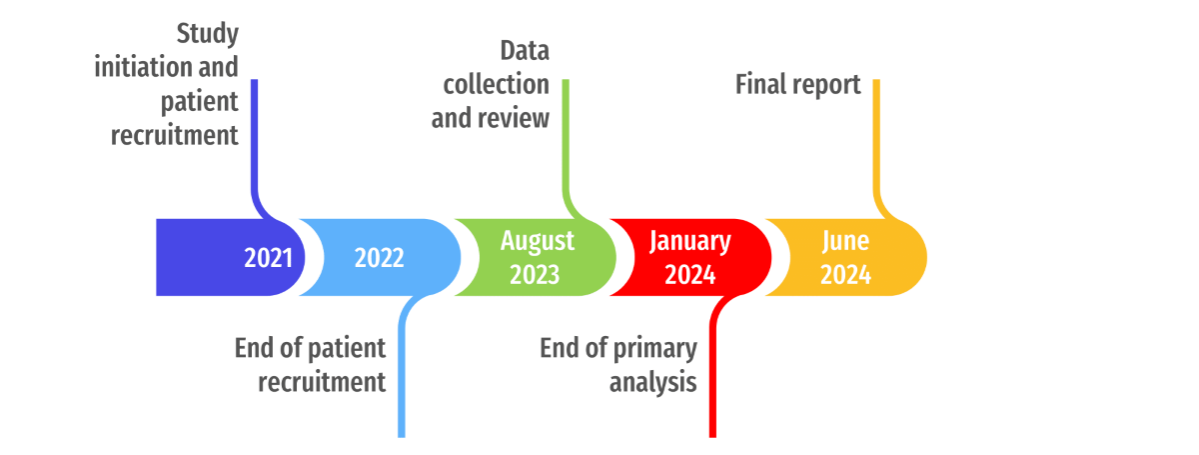
Timeline of the PHAROS trial.

A timeline of the PHAROS trial (Figure 2) illustrates each phase of the study, from patient recruitment through final analysis and reporting.

These timeline indicators and progress markers will enable ongoing tracking of study completion and ensure that each stage of data collection and analysis aligns with the study’s objectives.

### Statistical Methods

Continuous variables will be characterized as the mean with standard deviation. Categorical variables will be expressed as numbers and percentages.

To compare the number of drugs, active substances, and DRPs at hospital admission and discharge, the paired Wilcoxon signed-rank test will be used due to the non-normal distribution of differences between the 2 time points. Normality will be assessed with the Shapiro-Wilk test. To determine the effect size of the intervention, Cohen *d* will be calculated and categorized as small, medium, or large based on established intervals. Patients’ understanding of their pharmacotherapy at hospital admission will be evaluated using a 3-point scale. The average score per medication will be calculated, categorizing patients into groups based on their understanding: good (2-3 points per medication), modest (1-2 points per medication), and poor (0-1 point per medication).

## Discussion

### Importance

The PHAROS study is the first prospective clinical trial in Slovakia to evaluate pharmacist-led interventions on DRPs in vascular surgery patients. Our findings add to the growing body of evidence that pharmacist involvement can reduce the prevalence of DRPs, improve patient understanding of medications, and enhance overall pharmacotherapy outcomes for hospitalized patients.

### Comparison With Prior Work

Several studies have examined the role of pharmacists in preventing and managing DRPs across various clinical settings [57], including vascular surgery. Hohn et al [45] and Martínez López et al [51] documented a decrease in DRPs due to pharmacist-led interventions, reporting an average of 1.3 and 1.7 DRPs per patient, respectively. These studies support the role of pharmacists in reducing DRPs and improving medication safety in hospitalized patients. Findings from Schmelzer et al [58] and other international studies also demonstrated a significant reduction in DRPs at discharge.

The impact of pharmacist-led interventions on vascular surgery patients specifically has not been extensively studied. However, studies in other settings, such as those by Hohn et al [45] and Rychlíčková et al [59], have shown positive outcomes from pharmacist interventions. Singh et al [60] highlighted the underprescription of statins in patients with peripheral artery disease, attributing this to clinician-related barriers such as concerns about adverse effects. The PHAROS study aims to contribute to this literature by demonstrating that pharmacist involvement in MedRec and MedRev supports DRP management and optimizes pharmacotherapy, underscoring the importance of pharmacist intervention in promoting guideline-adherent care.

An important aspect of the PHAROS study is its focus on patient education. Based on the prior literature, educating patients about their medications is strongly associated with better adherence and a reduction in DRPs [31,32,61,62]. Including patient education as part of the pharmacist’s role is intended to support informed patient participation in their treatment.

### Challenges and Barriers

Despite the promising outcomes of pharmacist-led interventions, challenges remain in their implementation. One significant barrier is resistance to change among health care professionals, especially in settings where the role of pharmacists in medication management is not well established. As noted by Hohn et al [45], physicians may be hesitant to accept pharmacist recommendations, particularly in complex cases involving polypharmacy and comorbidities. Successful pharmacist-led interventions require clear protocols, comprehensive training, and effective collaboration with other health care professionals. The lack of legislative requirements for MedRec and MedRev in Slovakia demonstrates the need for policy changes to integrate pharmacists systematically into patient care transitions. This is particularly important, as international guidelines, such as those from the European Association of Hospital Pharmacists [63], emphasize the role of MedRec in ensuring safe and effective care.

### Future Directions

Building on the results of this pilot study, future research should seek to validate these findings through a larger, randomized controlled trial, which would allow for a more rigorous assessment of pharmacist-led interventions on DRPs. Such studies could incorporate a control group, a larger sample size, and postdischarge follow-up to evaluate long-term effects. Expanding research to diverse health care settings and patient demographics could provide insights into optimizing pharmacist interventions across various clinical contexts.

### Limitations of the Study

Our study has several limitations. First, it was conducted as a single-center trial and performed in specific departments and with specific indications; therefore, the results may not be applicable under different conditions. Second, the study did not include a control group. Although all investigators were trained in all study processes, some degree of subjectivity is possible in the assessment of DRPs. Moreover, the number of patients was limited. However, trained hospital pharmacists strengthened the methodology used in our trial.

### Conclusions

The study should determine that pharmaceutical care provided at hospital admission and at hospital discharge could reduce the prevalence rates of DRPs in our study setting. The pharmacist-led interventions upon hospital admission and discharge, followed by patient education, might be implemented in daily practice in health care in our hospital.

## Abbreviations

ADR: adverse drug reaction
ATC: Anatomical Therapeutic Chemical Classification
BPMH: best possible medication history
DRP: drug-related problem
HIS: hospital information system
MedRec: medication reconciliation
MedRev: medication review
PCNE: Pharmaceutical Care Network Europe Association
SQUIRE: Standards for Quality Improvement Reporting Excellence

## Data Availability

Only investigators from the National Institute of Cardiovascular Diseases have access to all patient data. They maintain an accurate and comprehensive list of biomedical research participants, along with the alphanumeric codes assigned to them, all documented in the “Participant Identification Sheet.” Both the completed Participant Identification Sheet and all research data are securely stored. Anonymized personal data are entered into the case report form in the online MIA document management system (DMS) database and are accessible only to investigators. The data sets used and analyzed during the study are available from the corresponding author upon reasonable request.
